# The Molecular Mechanisms of Cognitive Dysfunction in Long COVID: A Narrative Review

**DOI:** 10.3390/ijms26115102

**Published:** 2025-05-26

**Authors:** Elena Popa, Andrei Emilian Popa, Mihaela Poroch, Vladimir Poroch, Monica Iuliana Ungureanu, Ana Maria Slanina, Agnes Bacusca, Elena Adorata Coman

**Affiliations:** 1 Faculty of Medicine, “Grigore T. Popa” University of Medicine and Pharmacy, 16 Universitatii Str., 700115 Iasi, Romania; boanca.mihaela@umfiasi.ro (M.P.); vladimir.poroch@umfiasi.ro (V.P.); monica.ungureanu@umfiasi.ro (M.I.U.); ana_slanina@umfiasi.ro (A.M.S.); agnes.bacusca@umfiasi.ro (A.B.); elena.coman@umfiasi.ro (E.A.C.); 2 Department of Family Medicine, Preventive Medicine and Interdisciplinary, “Grigore T. Popa” University of Medicine and Pharmacy, Universitatii Str. 16, 700115 Iasi, Romania; 3 “Prof. Dr. Nicolae Oblu” Emergency Clinic Hospital, 700309 Iasi, Romania; andreiemilianpopa@gmail.com

**Keywords:** long-COVID, cognitive dysfunction, neuroinflammation

## Abstract

Cognitive dysfunction represents one of the most persistent and disabling features of Long COVID, yet its molecular underpinnings remain incompletely understood. This narrative review synthesizes current evidence on the pathophysiological mechanisms linking SARS-CoV-2 infection to long-term neurocognitive sequelae. Key processes include persistent neuroinflammation, blood–brain barrier (BBB) disruption, endothelial dysfunction, immune dysregulation, and neuroendocrine imbalance. Microglial activation and cytokine release (e.g., IL-6, TNF-α) promote synaptic dysfunction and neuronal injury, while activation of inflammasomes such as NLRP3 amplifies CNS inflammation. Vascular abnormalities, including microthrombosis and BBB leakage, facilitate the infiltration of peripheral immune cells and neurotoxic mediators. Hypothalamic–pituitary–adrenal axis dysfunction and reduced vagal tone further exacerbate systemic inflammation and autonomic imbalance. Biomarkers such as GFAP, NFL, IL-6, and S100B have been associated with both neuroinflammation and cognitive symptoms. Notably, transcriptomic signatures in Long COVID overlap with those observed in Alzheimer’s disease, highlighting shared pathways involving *tau* dysregulation, oxidative stress, and glial reactivity. Understanding these mechanisms is critical for identifying at-risk individuals and developing targeted therapeutic strategies. This review underscores the need for longitudinal research and integrative biomarker analysis to elucidate the molecular trajectory of cognitive impairment in Long COVID.

## 1. Introduction

Long COVID, also referred to as post-acute sequelae of SARS-CoV-2 infection (PASC), is a chronic, multisystemic condition that persists beyond the resolution of the acute phase of COVID-19. It is defined by the persistence or emergence of symptoms for at least 12 weeks following infection with SARS-CoV-2, in the absence of an alternative diagnosis [1,2]. While the manifestations of Long COVID are highly heterogeneous, cognitive dysfunction has emerged as one of the most frequent and debilitating symptoms, substantially impairing quality of life and functional independence [3,4,5].

This constellation of cognitive symptoms is commonly described by patients as “brain fog” and encompasses memory deficiencies, reduced attention span, slowed information processing, and executive dysfunction [5,6]. Notably, these impairments have been reported even in individuals with mild or asymptomatic acute infection and may persist for months after viral clearance [6,7]. Objectively, cognitive deficits have been identified through neuropsychological testing, including impairments in working memory, verbal fluency, and complex task execution, with implications for occupational performance and social functioning [8].

Although some features of Long COVID resemble post-viral syndromes described after infections such as Epstein–Barr virus or influenza, the global scale of the COVID-19 pandemic and the high burden of neurological symptoms make this condition a unique clinical and public health challenge [9,10]. The exact pathophysiological mechanisms underlying post-COVID cognitive dysfunction remain incompletely understood. However, emerging evidence points toward a convergence of neuroinflammatory, vascular, metabolic, and neurodegenerative pathways, which may collectively contribute to sustained neuronal dysfunction [10,11,12,13].

Given the complexity of the clinical phenotype and the diversity of the proposed mechanisms, an integrative approach is necessary to synthesize current findings. This article presents a narrative review based on a comprehensive literature search, integrating clinical and experimental data to explore the molecular mechanisms underlying cognitive impairment in Long COVID.

## 2. Materials and Methods

A comprehensive search was performed in PubMed, Scopus, and Web of Science for peer-reviewed articles published in the past five years. Additional sources were retrieved from the World Health Organization (WHO) and the Centers for Disease Control and Prevention (CDC). Search terms included “Long COVID”, “cognitive dysfunction”, and “neuroinflammation”. The reference lists of selected articles were also screened to identify further relevant studies. Out of approximately 1200 records initially identified, 169 sources were included in the final synthesis. These included 75 original research articles (including 52 observational cohorts, 20 longitudinal studies, and 3 clinical trials), 32 mechanistic investigations in animal or cellular models, 61 peer-reviewed reviews and meta-analyses with novel translational insights, and one public health reports from the WHO. Most studies originated from Europe, North America, and Asia, with limited data from low-income regions. Studies were considered eligible if they were published in English, peer-reviewed, and focused specifically on neurocognitive outcomes, mechanistic underpinnings, or biomarkers of cognitive dysfunction in the context of post-acute sequelae of SARS-CoV-2 infection. Editorials, commentaries, case reports, preprints, and studies unrelated to neurocognitive outcomes were excluded. Priority was given to original research articles, including observational cohorts, longitudinal investigations, and mechanistic studies. Review articles were included only if they contained new quantitative syntheses (e.g., systematic reviews or meta-analyses) or filled specific conceptual gaps not directly covered by primary studies. Given the narrative format of this review, no formal risk of bias or quality assessment tools were applied. Instead, a thematic synthesis approach was adopted to integrate and categorize the findings on the basis of methodological clarity, scientific rigor, and relevance to the neurobiological mechanisms of Long COVID-related cognitive impairment.

## 3. Neurological and Cognitive Manifestations of Long COVID

Long COVID manifests itself through a wide range of neurological symptoms that affect both the central and peripheral nervous systems. These can occur during acute infection or later, persisting with varying intensity. Common manifestations include fatigue, headache, anosmia, ageusia, dizziness, myalgia, neuropathic pain, and neuropsychiatric disorders such as depression, anxiety, insomnia, and irritability [5,6,7,10].

Among the most disabling sequelae are cognitive disorders, often referred to as “brain fog” [11,12,13,14], which include mental confusion, memory loss, and concentration disorders [15,16,17,18,19]. Objective assessments, including the Montreal Cognitive Assessment (MoCA), the Mini-Mental State Examination (MMSE), and the Symbol Digit Modalities Test (SDMT), have confirmed impairments in attention, executive function, verbal fluency, and working memory [20,21,22]. Prevalence estimates vary, with meta-analyses reporting rates of 20–26% at 3–12 months after infection [3,11], and longitudinal data indicating increases from 16% to 26% between two months and one year [23]. A population-based study of over 1.3 million people found persistent neurocognitive sequelae up to two years after infection [1,24]. These impairments significantly affect daily functioning and occupational performance [25,26,27].

Recent systematic reviews have expanded current knowledge regarding the persistence of neurocognitive impairments in individuals affected by Long COVID. One analysis conducted in 2024 included 36 studies and highlighted lasting deficits in executive functions, memory, attention, and processing speed [14]. The authors emphasized the importance of implementing targeted interventions to alleviate these symptoms. Another meta-analysis from 2025, which synthesized data from 33 studies, reported a significantly elevated risk of experiencing memory problems and difficulties with concentration at least four weeks following SARS-CoV-2 infection [28]. Together, these findings reinforce the widespread nature of cognitive symptoms in the post-acute phase of COVID-19 and the need for systematic follow-up.

Additional evidence from recent large-scale cohort studies supports these observations. One observational study from 2024, involving more than 140,000 participants, identified persistent cognitive impairments, particularly in memory and executive function, among individuals reporting long-term symptoms [29]. Notably, these deficits remained even after other physical symptoms had resolved. Another study from the same year reported significant cognitive slowing in patients with Long COVID compared with matched control groups, on the basis of standardized neuropsychological testing [30]. These findings emphasize the enduring cognitive consequences of Long COVID and the pressing need for sustained clinical and research engagement in this field.

Emerging evidence suggests that psychosocial stress may exacerbate *brain fog* through molecular mechanisms involving neuroendocrine imbalance, systemic inflammation, and blood–brain barrier (BBB) dysfunction. High levels of psychological stress activate the hypothalamic–pituitary–adrenal (HPA) axis, leading to dysregulated cortisol secretion and increased expression of proinflammatory cytokines such as interleukin-6 (IL-6) and tumor necrosis factor alpha (TNF-α) [14,16,31,32]. These mediators contribute to glial activation, oxidative stress, impaired synaptic plasticity, and disruption of BBB integrity [33,34,35]. Taken together, these processes have been implicated in the pathogenesis of cognitive and neuropsychiatric symptoms associated with Long COVID [6,9,10]. Furthermore, a large-scale online survey conducted in Romania identified high rates of anxiety, depression, and psychological distress during the pandemic, with potential implications for the persistence of post-infection cognitive and emotional symptoms [36]. These observations underscore the importance of multidisciplinary management strategies, including psychoneuroimmunological assessment, to address the complex interaction between molecular and psychosocial factors contributing to cognitive dysfunction in Long COVID [37,38].

It is important to note that cognitive symptoms often occur independently of mood disorders, with several studies demonstrating their persistence even after controlling for depression, anxiety, or fatigue [36,39]. Remarkably, individuals with mild or asymptomatic infections may still develop long-term cognitive impairment, suggesting a potential role for direct viral invasion or immune-mediated central nervous system (CNS) damage [40,41].

Converging molecular and neurofunctional mechanisms have been proposed to underlie the cognitive overlap between Long COVID and post-concussion syndrome. Comparative studies have identified common clinical features such as attention deficit, sensory hypersensitivity, fatigue, and anxiety. These symptoms are thought to result from diffuse neural network dysfunction, possibly driven by glial activation, neuroinflammation, and impaired synaptic plasticity—mechanisms also described in mild traumatic brain injury. Evidence suggests that both conditions may involve the transient disruption of connectivity, altered neuromodulation, and BBB dysfunction, which together underpin cognitive deficits even in the absence of obvious structural brain damage. This information may support the adaptation of cognitive rehabilitation strategies from post-traumatic models to Long COVID [42].

Other neurological features include hearing loss, tinnitus, vestibular dysfunction (e.g., vertigo), paresthesia, and autonomic disorders such as postural orthostatic tachycardia syndrome (POTS) [43,44,45]. The variability and late onset of these manifestations, combined with the absence of standardized diagnostic criteria, contribute to their frequent under recognition. Neuroimaging studies have revealed structural and functional abnormalities, including hippocampal atrophy, reduced gray matter volume, and changes in connectivity within cognitive networks [20,46,47,48].

Overall, neurological and cognitive manifestations constitute a significant but often underdiagnosed aspect of post-viral morbidity. Their persistence and functional burden highlight the urgent need for systematic assessment and a mechanistic understanding to inform effective therapeutic strategies.

## 4. Pathogenic Mechanisms Underlying Neurological Dysfunction

**Figure 1:**
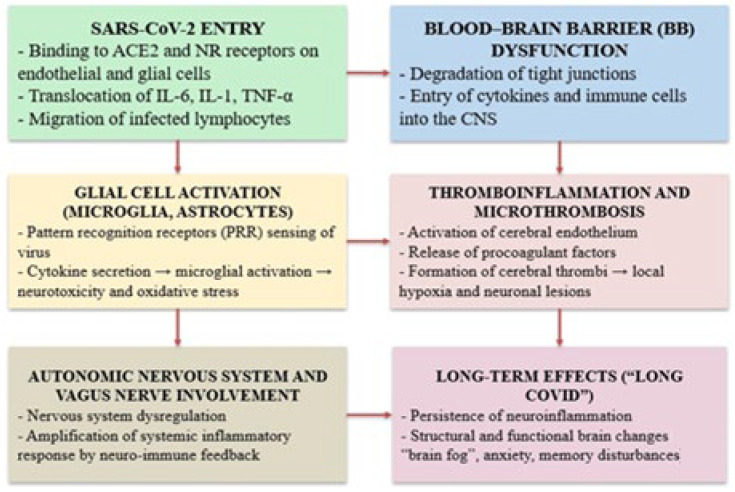
Mechanisms of cognitive dysfunction in Long COVID.

Neurological manifestations in Long COVID stem from a multifactorial pathophysiological process involving both systemic and central nervous system disruptions. Key interconnected pathways—including neuroinflammation, BBB disruption, endothelial dysfunction, microvascular injury, and persistent immune activation—contribute to synaptic damage, glial reactivity, and impaired cerebral perfusion [49,50,51,52,53,54,55,56] (Figure 1).

This diagram illustrates the main biological mechanisms involved in the development of cognitive and neuropsychiatric symptoms after SARS-CoV-2 infection. The virus enters through the angiotensin-converting enzyme 2 (ACE2) and neuropilin-1 (NRP1) receptors on endothelial and glial cells, triggering a series of events, including the systemic release of cytokines such as interleukin-6 (IL-6), interleukin-1 beta (IL-1β), and tumor necrosis factor alpha (TNF-α) and the infiltration of immune cells into the central nervous system (CNS). These processes promote disruption of the blood–brain barrier (BBB), activation of glial cells (microglia and astrocytes), and thromboinflammatory responses, including cerebral microthrombosis. The resulting pathophysiological changes lead to neuroinflammation, oxidative stress, and neuronal injury. In parallel, dysregulation of the autonomic nervous system (ANS) and the vagus nerve exacerbates inflammation through neuroimmune feedback. Together, these mechanisms contribute to the long-term sequelae of Long COVID, including persistent neuroinflammation, cognitive dysfunction (“brain fog”), anxiety, and memory impairment [51,53,54,55,56].

The following section discusses the molecular and cellular processes through which SARS-CoV-2 may alter brain function in the context of Long COVID, offering insights into the biological basis of persistent cognitive symptoms.

### 4.1. Neuroinflammation and Its Impact on Brain Function

**Figure 2:**
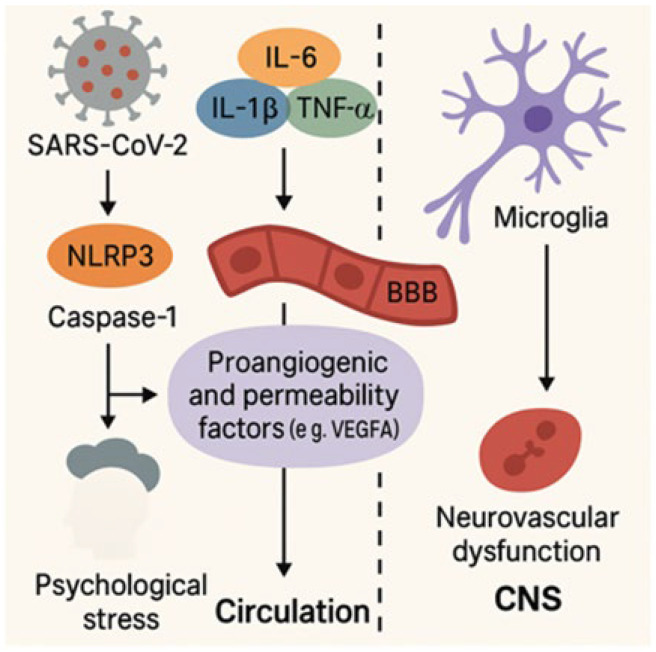
Neuroinflammatory and vascular pathways in Long COVID.

Neuroinflammation has emerged as a central mechanism contributing to the cognitive impairment observed in Long COVID, reflecting a sustained and maladaptive immune activation within the CNS [48]. As illustrated in Figure 2, this pathological process involves complex interactions among peripheral immune signals, microglial activation, and blood–brain barrier dysfunction, ultimately leading to neuronal dysfunction and cognitive decline [35,54,55,57,58,59].

Figure 2 illustrates the interplay between inflammatory and vascular processes triggered by SARS-CoV-2 infection. Activation of the NLRP3 (NOD-like receptor family pyrin domain containing 3) inflammasome and caspase-1 promotes the release of proinflammatory cytokines, including interleukin-6 (IL-6), interleukin-1 beta (IL-1β), and tumor necrosis factor alpha (TNF-α). These cytokines upregulate vascular permeability and proangiogenic factors such as vascular endothelial growth factor A (VEGF-A), which disrupt the blood–brain barrier (BBB) and alter vascular homeostasis. This cascade facilitates microglial activation and contributes to neurovascular dysfunction, ultimately compromising central nervous system (CNS) integrity in Long COVID [35,54,55,57,58,59].

Although the CNS has traditionally been considered immunologically privileged [60], evidence now shows that systemic infections, including SARS-CoV-2, can trigger robust neuroimmune activation [33]. This includes direct viral interactions with neural cells and sustained peripheral inflammation that affects the CNS through a compromised BBB [61,62]. Microglia, the resident immune cells of the CNS, respond to systemic cytokines and damage-associated signals, such as the adenosine triphosphate (ATP) released from injured neurons, by shifting into an activated phenotype [62,63]. Chronic microglial activation leads to the release of proinflammatory cytokines (e.g., TNF-α, IL-6), reactive oxygen species (ROS), and complement components that promote synaptic loss, demyelination, and neuronal injury [64,65,66,67]. Astrocytes and microglia contribute to CNS damage by secreting nitric oxide, glutamate, and prostaglandins, amplifying neurotoxicity [68,69].

A central role is played by intracellular inflammasomes, particularly NOD-, LRR- and pyrin domain-containing protein 3 (NLRP3), which mediate the production of interleukin-1 beta (IL-1β) and interleukin-18 (IL-18), which are cytokines implicated in neurodegenerative diseases and COVID-19-related brain injury [54,70]. Upon activation, NLRP3 promotes caspase-1-dependent cytokine maturation, thereby sustaining neuroinflammation and contributing to cognitive dysfunction. Innate immune sensors, such as Toll-like receptors (TLRs) and NOD-like receptors (NLRs), expressed on glial and neuronal cells, recognize viral motifs and amplify inflammation via cytokine and complement activation [54,61,66,69]. For example, the chemokine CCL11 (eotaxin-1), which is elevated in the cerebrospinal fluid (CSF) of Long COVID patients, inhibits neurogenesis and oligodendrocyte function, further promoting cognitive decline [71].

**Figure 3:**
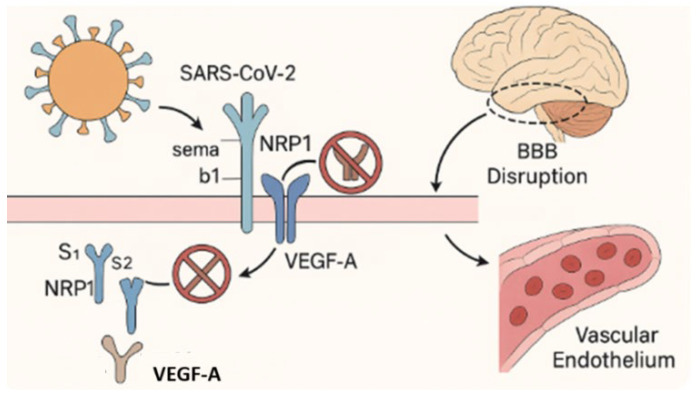
Disruption of the VEGF-A/NRP1 signaling axis by SARS-CoV-2.

Endothelial dysfunction induced by direct viral infection and inflammation contributes to neuroinflammation and cognitive impairment through mechanisms including microvascular thrombosis, BBB disruption, and tissue hypoxia [11,72,73]. SARS-CoV-2 can traverse the BBB by infecting angiotensin-converting enzyme2 (ACE2)-expressing endothelial cells and pericytes, or via transcytosis, enabling viral entry into the CNS [11,74]. Neuropilin-1 (NRP1), co-expressed with ACE2 in olfactory and vascular endothelial tissues, facilitates CNS viral entry and regulates the vascular endothelial growth factor A (VEGF-A)-dependent pathways involved in angiogenesis and vascular permeability [55,74]. Disruption of the NRP1/VEGF-A axis by the viral spike protein exacerbates vascular instability and has been associated with anosmia, nociceptive alterations, and increased vulnerability to neuroinflammatory damage [51,55,75,76] (Figure 3).

This figure illustrates the disruption of neuropilin-1 (NRP1)-mediated signaling by SARS-CoV-2. Under physiological conditions, NRP1 functions as a co-receptor for vascular endothelial growth factor A (VEGF-A, labeled “A”) and semaphorin b1 (Sema b1), coordinating the signaling pathways involved in vascular homeostasis, blood–brain barrier (BBB) stability, and neuronal guidance [55,75]. These ligands bind to the b1 domain of NRP1 to maintain endothelial integrity and regulate neurovascular function. Following SARS-CoV-2 infection, the S1 subunit of the viral spike protein competitively binds to the same b1 domain of NRP1, blocking access to endogenous ligands. This interference disrupts intracellular signaling, compromises BBB function, promotes endothelial dysfunction, and contributes to the neurovascular injury and impaired CNS homeostasis observed in Long COVID [55,75,76].

MicroRNAs (miRNAs) and extracellular vehicles (EVs) are additional contributors to the neuroinflammatory environment. miR-24, a suppressor of NRP1 [77], has demonstrated anti-inflammatory and anti-viral effects and is a potential modulator of BBB integrity in Long COVID [78]. Molecular analyses have revealed the presence of SARS-CoV-2 proteins and injury markers, such as glial fibrillary acidic protein (GFAP) and agrin, in astrocyte- and neuron-derived exosomes (ADEVs and NDEVs), even in individuals with mild acute COVID-19, indicating persistent cellular dysfunction [51,58,69,79]. These extracellular vesicles (EVs) also carry microRNAs (miRNAs), such as miR-146a, miR-21, and miR-155, which regulate glial activation and oxidative stress via nuclear factor kappa-light-chain-enhancer of activated B cells (NF-κB) signaling and may contribute to impaired neurogenesis and synaptic plasticity [80].

Persistent elevation of cytokines such as IL-6, IL-1β, and TNF-α has been consistently observed in Long COVID and correlates with cognitive symptoms and brain abnormalities [14,16,81,82]. TNF-α is a central mediator of microglia-driven synaptic loss, as shown in experimental viral encephalitis models and prospective cohort studies in humans [48,83,84]. Biomarkers such as neurofilament light chain (NFL), GFAP, and galectin-3 (LGALS3) are elevated in the CSF and serum of affected individuals, indicating ongoing astrocytic and axonal injury [33,74,82]. These markers correlate with disease severity and cognitive dysfunction [58,82]. Systemic hyperinflammation—indicated by persistently elevated TNF-α and IL-6 levels in plasma and CSF—has been associated with microstructural brain abnormalities and glial activation [84,85].

In some cases, infection-triggered autoimmunity contributes to CNS pathology. Autoantibodies targeting cytokines, phospholipids, and neural antigens have been identified in Long COVID and are associated with neuropsychiatric symptoms and vascular complications [10,27,86,87]. Therapeutic strategies targeting inflammatory mediators have been explored. IL-6 receptor antagonists, particularly tocilizumab, have demonstrated survival benefits in severe cases of COVID-19, according to a large meta-analysis of randomized controlled trials [88]. Given the persistence of IL-6 pathway activation in convalescent COVID-19 patients [89], their potential application in Long COVID is currently under investigation. In contrast, TNF-α inhibitors are currently undergoing clinical evaluation, with concerns remaining regarding their safety and therapeutic specificity in the context of post-acute COVID-19 [90,91].

### 4.2. Blood–Brain Barrier Disruption and Neurovascular Injury in Long COVID

Vascular dysfunction plays a central role in COVID-19’s pathogenesis, contributing to systemic and neurological complications in Long COVID [92]. Disruption of the BBB—a regulatory interface composed of endothelial cells, pericytes, astrocytes, and basal lamina—permits peripheral cytokines and neurotoxins to infiltrate the CNS (Figure 2), triggering neuroinflammation and cognitive impairment [35,93]. Neuropathological investigations have revealed vascular abnormalities characteristic of COVID-19, including microthrombosis, small vessel vasculitis, and diffuse endothelial dysfunction. These findings support the hypothesis that acute vascular injury may serve as a precursor to long-term cognitive impairment, particularly through mechanisms involving microcirculatory disorders and neuroinflammation [94].

SARS-CoV-2 binds to ACE2-expressing endothelial cells, pericytes, and astrocytes, following spike (S) protein priming by transmembrane protease serine 2 (TMPRSS2) and furin, which together facilitate viral entry [95,96]. Once internalized, the viral components activate glial cells, amplify cytokine cascades, and destabilize the BBB [46,93]. A characteristic cytokine profile, including IL-1β, IL-6, IL-10, interferon gamma (IFN-γ), monocyte chemoattractant protein-1 (MCP-1), and granulocyte–macrophage colony-stimulating factor (GM-CSF), enhances endothelial permeability via apoptosis, junctional breakdown, and non-canonical NF-κB signaling in microglia [86,97].

Astrocytic release of TNF-α, nitric oxide, glutamate, and ROS exacerbates vascular and neuronal injury. Complement activation and coagulation imbalance contribute further to microvascular disruption [98]. Additionally, the kallikrein–kinin system (KKS), through bradykinin accumulation, enhances vascular leakage via stimulation of bradykinin receptors B1 and B2, a mechanism that is particularly relevant in vulnerable brain regions [35,51].

Hypoxia secondary to respiratory failure also compromises blood–brain barrier (BBB) function by inducing vascular endothelial growth factor (VEGF) signaling, oxidative stress, and hypoxia-inducible factor 1-alpha (HIF-1α)-dependent modulation of ACE2 [99,100]. Elevated matrix metalloproteinase-9 (MMP-9) degrades Type IV collagen in the basal lamina, impairing both the BBB and the blood–CSF barrier [35,101].

Multiple BBB invasion pathways have been proposed: a transcellular route via ACE2-mediated entry, and a paracellular route involving RhoA-driven cytoskeletal contraction and tight junction disassembly [35,97,100,101]. Hematogenous dissemination and transcytosis of infected monocytes and immune cells may also contribute to viral neuroinvasion [61].

Clinical and postmortem studies have revealed elevated CSF albumin, fibrinogen extravasation, and microvascular pathology in brain tissue, consistent with barrier breakdown [102,103]. A cross-sectional study by Greene et al. demonstrated persistent BBB disruption up to 211 days post-infection in Long COVID patients with cognitive symptoms [78]. Magnetic Resonance Imaging (MRI) findings included cortical thinning, increased CSF volume, and white matter loss in the frontal and temporal lobes [92]. Concurrently, serum markers (S100B, IL-6, IL-13, basic fibroblast growth factor (bFGF), and MCP-1) were significantly elevated, alongside transcriptomic profiles showing upregulated transforming growth factor beta (TGF-β) signaling and T cell activation [92].

Functionally, sera from Long COVID patients induced endothelial activation and expression of TNF and adhesion molecules, such as ICAM-1 and VCAM-1, in brain endothelial cell cultures [92,104]. Exposure to recombinant S1 protein also disrupted tight junction integrity, reflecting neuropathological observations of perivascular inflammation and microvascular injury [105].

Notably, the temporal lobes, linked to both olfaction and cognition, exhibited the most consistent structural and functional disruption, potentially explaining the clinical correlation between acute-phase anosmia and subsequent cognitive dysfunction [106,107].

In addition to canonical inflammatory mediators, elevated TGF-β has been identified in Long COVID patients with cognitive symptoms, supporting its dual role in BBB permeability and neurodegeneration. This parallels findings in chronic fatigue syndrome, a condition with overlapping neurocognitive features [92,106,108].

Together, persistent endothelial activation, immune dysregulation, and spike protein-mediated barrier injury define a core mechanism driving cognitive impairment in Long COVID. The BBB thus emerges as both a marker of disease progression and a potential therapeutic target.

### 4.3. Endothelial Dysfunction and Cerebral Microvascular Thrombosis in Long COVID

Endothelial dysfunction is a key driver of Long COVID pathology, particularly within the CNS [109,110]. Cerebral endothelial cells maintain vascular homeostasis and BBB integrity [111,112] but are disrupted by SARS-CoV-2 through direct ACE2-mediated entry and inflammation-induced activation, leading to increased permeability and immune infiltration [110,112,113].

BBB disruption facilitates neuroinflammation and promotes a pro-thrombotic state. Activated ECs upregulate adhesion molecules such as ICAM-1, VCAM-1, and selectins, supporting leukocyte adhesion and platelet aggregation [110,113]. These events set the stage for cerebral microthrombosis, compromising oxygen delivery to neurons and glia. Neuropathological analyses have confirmed widespread microvascular injury and microthrombi in the brains of Long COVID patients, associated with mitochondrial dysfunction, local hypoxia, and neuronal apoptosis—mechanisms contributing to symptoms such as *brain fog*, impaired memory, and reduced attention span [46,114,115].

Advanced neuroimaging studies, including dynamic contrast-enhanced MRI, reveal increased BBB permeability and perfusion deficits, especially in the frontal and temporal lobes—regions involved in memory and executive function [35]. These findings are paralleled by persistent hypercoagulability, with circulating microclots detected weeks to months after recovery [102,110]. Microthrombi are thought to obstruct small cerebral vessels in metabolically active areas like the hippocampus and prefrontal cortex, impairing synaptic homeostasis [50]. Histological studies support sustained ischemic injury, including apoptotic neuronal loss and mitochondrial fragmentation [49].

Inflammation-induced endothelial activation plays a central role in this process. The COVID-19-associated cytokine storm, marked by elevated IL-6, IL-1β, TNF-α, and related mediators, upregulates tissue factor and von Willebrand factor expression, while downregulating anticoagulant pathways such as thrombomodulin and protein C [106,116,117]. Persistently elevated D-dimer and fibrinogen levels have been reported months after infection, indicating ongoing thrombin generation and vascular stress [116,117,118].

Beyond cognitive symptoms, cerebral microthrombosis has been implicated in stroke, transient ischemic attacks (TIAs), and progressive neurocognitive decline. Although often undetectable with standard neuroimaging, the cumulative burden of microclots likely contributes to chronic hypoperfusion and neuroinflammatory injury [50,119]. In parallel, oxidative stress, nitric oxide (NO) depletion, and impaired vasodilation exacerbate endothelial dysfunction and further compromise cerebral perfusion [120].

In addition to classical inflammatory mediators, metabolic regulators involved in vascular homeostasis have also emerged as key players in post-COVID endothelial pathology. A recent study analyzing peripheral immune cells from patients with metabolic syndrome—a condition frequently overlapping with Long COVID—demonstrated a significant reduction in peroxisome proliferator-activated receptor alpha (PPARα) expression in eosinophils, which was inversely correlated with CD36 receptor levels [121]. PPARα acts as a transcriptional regulator that attenuates inflammatory gene expression and promotes lipid oxidation, thus protecting against endothelial activation. Conversely, increased CD36 expression facilitates the uptake of oxidized lipids, foam cell formation, and activation of the NLRP3 inflammasome—features of a proinflammatory and pro-thrombotic vascular state [121,122].

Importantly, CD36 was recently shown to mediate SARS-CoV-2 envelope protein-induced platelet activation and thrombosis, directly linking viral elements to thromboinflammatory pathways [123]. This insight suggests that CD36 overexpression may not only reflect metabolic dysregulation but also actively contributes to virus-induced microvascular injury in Long COVID. The inverse expression pattern of PPARα and CD36 thus represents a shift toward endothelial dysfunction, oxidative stress, and immunothrombosis—factors that likely perpetuate the multisystem impairments observed in this syndrome. These findings support the exploration of metabolic–inflammatory pathways as potential therapeutic targets.

Circulating endothelial-derived microparticles and soluble markers such as ICAM-1, VCAM-1, and E-selectin have also been found to be elevated in Long COVID and correlate with disease severity and cognitive dysfunction [124,125]. These may serve as accessible biomarkers for monitoring cerebral microvascular involvement and tailoring therapeutic strategies.

Taken together, endothelial dysfunction and cerebral microthrombosis, sustained by chronic inflammation, immune imbalance, and coagulopathy, appear to be central drivers of neurovascular injury and cognitive impairment in Long COVID.

### 4.4. Neuroendocrine Dysregulation and Autonomic Nervous System Dysfunction in Long COVID

An expanding body of evidence implicates dysregulation of the autonomic–immune–endocrine axis in the persistent neurological and systemic symptoms of Long COVID [126]. Vagal dysfunction, central to the cholinergic anti-inflammatory pathway (CAP), contributes to dysautonomia, manifesting as POTS, fatigue, orthostatic intolerance, and palpitations [127,128].

SARS-CoV-2 has been detected in the vagus nerve, which expresses ACE2, neuropilin-1, and TMPRSS2 along its trajectory and at its brainstem entry point [129,130,131]. Neuroinvasion may induce chronic local inflammation and reduced vagal tone, impairing the CAP and sustaining a systemic proinflammatory state, a hallmark of inflammaging—particularly relevant in older adults with immune senescence [131].

A reliable surrogate of vagal tone is heart rate variability (HRV), which reflects central–peripheral autonomic regulation and neurovisceral integration [132]. Decreased HRV correlates with elevated IL-6, linking vagal dysfunction to inflammation and aging-related immune imbalance [133]. HRV is thus emerging as a predictive biomarker for post-COVID dysautonomia and associated cardiovascular and neurocognitive risks [134].

Additional markers of autonomic and neuroendocrine imbalance include catecholamines (dopamine, norepinephrine), serotonin, and cortisol. Chronic inflammation in Long COVID diverts tryptophan metabolism toward the kynurenine pathway, depleting peripheral serotonin—a process that may underlie mood disturbances, memory impairment, and reduced vagal tone. Similarly, dopamine deficiency may contribute to low motivation and executive dysfunction [135,136,137,138,139].

Among autonomic disorders, POTS is the most frequently reported cardiovascular manifestation after SARS-CoV-2 infection. A European survey across specialty centers found that over 60% of clinicians attributed new-onset POTS to prior COVID-19 [140]. Other dysautonomic features include orthostatic hypotension and vasovagal syncope. Although pathogenesis is multifactorial, encompassing baroreflex failure, neurotropism, and autoimmunity, an immune-mediated mechanism is strongly implicated. Notably, post-vaccination POTS is rare, typically milder, and lacks consistent causality [141]. Management includes non-pharmacologic measures (hydration, salt, compression garments, graded exercise) and medications (propranolol, fludrocortisone, midodrine, pyridostigmine, ivabradine, clonidine) in refractory cases [141].

Beyond autonomic nervous system (ANS) dysfunction, Long COVID is associated with persistent hypocortisolism and hypothalamic–pituitary–adrenal (HPA) axis suppression [129,130]. Reduced serum cortisol levels, often in the absence of compensatory adrenocorticotropic hormone (ACTH) elevation, suggest hypothalamic or pituitary dysfunction [142,143,144,145]. This hormonal pattern mirrors that seen in myalgic encephalomyelitis/chronic fatigue syndrome, supporting shared neuroendocrine mechanisms [146,147].

The persistence of hypocortisolism beyond 12 months raises concerns about long-term HPA axis dysfunction [142]. Proposed mechanisms include hypothalamic or pituitary inflammation, adrenal injury, and glucocorticoid receptor resistance—all contributing to impaired feedback regulation and hormonal imbalance [142,147,148]. These alterations may drive chronic fatigue, affective symptoms, and cognitive dysfunction in Long COVID.

Collectively, these findings support a model of systemic dysregulation involving the ANS, HPA axis, and inflammatory pathways, which reinforces a proinflammatory state and reduces physiological resilience. In this context, mineral imbalances such as hypomagnesemia may further exacerbate systemic and neurological symptoms. Recent data have highlighted a significant association between low magnesium levels and persistent manifestations such as fatigue, cognitive impairment, myalgia, and dysautonomia in Long COVID patients. Magnesium stabilizes neuronal membranes, modulates calcium signaling, and attenuates neurogenic inflammation, thereby potentially exerting a neuroprotective effect. Thus, assessment and correction of magnesium levels may represent a valuable supportive measure in the management of neuropsychiatric and muscular symptoms in Long COVID [149]. Addressing such modifiable factors is particularly relevant, as this triad of autonomic, endocrine, and inflammatory dysregulation promotes inflammaging, increases the risk of neurocognitive decline, and sustains multisystem symptoms. Biomarkers such as HRV, cortisol, serotonin, dopamine, and circulating cytokines (e.g., IL-6, IL-17, TNF-α) may function as diagnostic tools, prognostic indicators, and therapeutic targets, supporting personalized approaches to restore autonomic and neuroendocrine balance in Long COVID.

## 5. Converging Pathogenic Mechanisms Between Long COVID and Alzheimer’s Disease

In contrast to classical neurodegenerative disorders such as Alzheimer’s or Parkinson’s disease, the neuropathological mechanisms associated with SARS-CoV-2 infection demonstrate a unique interplay among systemic immune activation, endothelial dysfunction, and direct neuroinvasion. While tau phosphorylation, oxidative stress, and mitochondrial impairment are common features, SARS-CoV-2 elicits an exaggerated NLRP3 inflammatory response and widespread cytokine release (e.g., IL-1β, IL-6), which disrupts the blood–brain barrier (BBB) and activates glial cells [33,54,69,70,71]. Moreover, viral entry via neuropilin-1 and ACE2 receptors on the olfactory neurons and brain endothelial cells bypasses classical synaptic transmission, potentially explaining the rapid onset of cognitive dysfunction and structural brain changes seen in Long COVID [5,74,75,104,106,109]. Dysregulation of the hypothalamic–pituitary–adrenal (HPA) axis and persistent microglial activation further differentiate Long COVID from chronic, slowly progressive neurodegenerative disorders [129,130,143,144,150].

**Figure 4:**
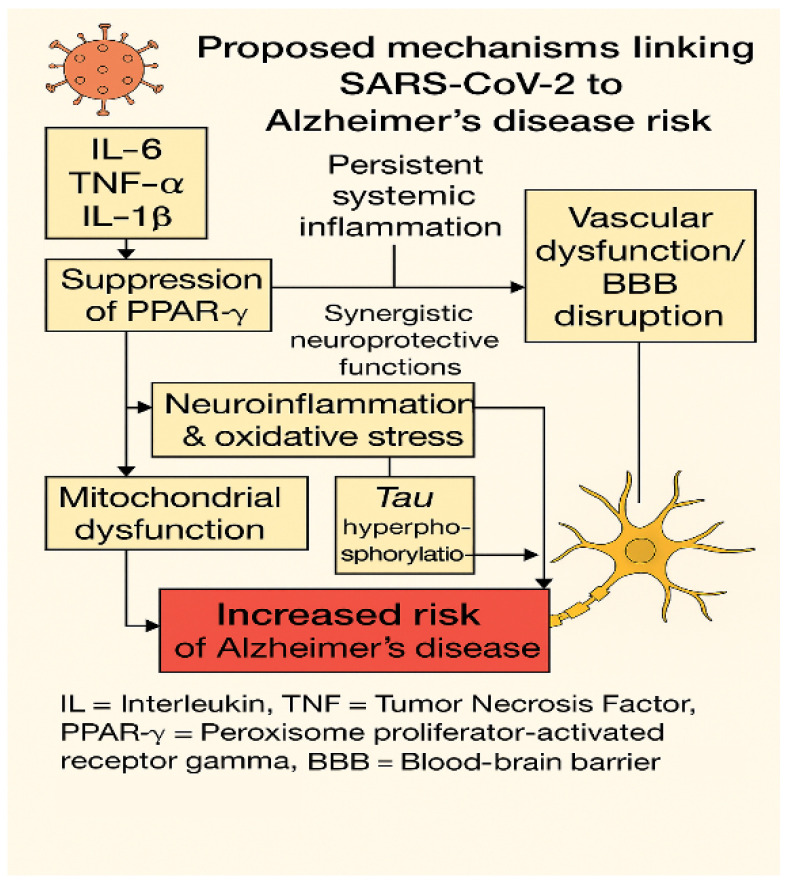
Molecular crosstalk between Long COVID and Alzheimer’s disease pathogenesis.

These distinct and overlapping features are illustrated in Figure 4, which summarizes the molecular and cellular mechanisms linking SARS-CoV-2 to Alzheimer’s-like neurodegeneration.

This diagram illustrates the main molecular pathways linking SARS-CoV-2 infection to Alzheimer’s-like neurodegeneration, as observed in Long COVID. Persistent systemic inflammation, characterized by elevated circulating levels of interleukin-6 (IL-6), tumor necrosis factor alpha (TNF-α), and interleukin-1 beta (IL-1β), leads to suppression of two key neuroprotective transcription factors: peroxisome proliferator-activated receptor gamma (PPAR-γ) and nuclear factor erythroid 2–related factor 2 (NRF2) [31,51,151]. These factors function synergistically to preserve mitochondrial integrity, redox balance, and vascular homeostasis [152,153]. Their downregulation promotes oxidative stress and mitochondrial dysfunction, which, together with activation of kinases such as GSK-3β and CDK5, contributes to tau hyperphosphorylation—an early neuropathological feature of Alzheimer’s disease [82,154].

In parallel, endothelial injury and disruption of the blood–brain barrier (BBB) facilitate the central nervous system’s exposure to peripheral cytokines, oxidative mediators, and neurotoxic molecules, thereby amplifying neuroinflammatory responses [35,73,106]. Notably, the PPAR-γ–NRF2 relationship reflects a functional synergy rather than a sequential pathway, indicating integrated regulation of anti-inflammatory and antioxidant responses [151,152]. Altogether, this interconnected cascade may underlie the increased vulnerability to Alzheimer’s-type cognitive decline in individuals with predisposing genetic, metabolic, or vascular risk profiles.

Despite its distinctive pathophysiological features, increasing evidence suggests that SARS-CoV-2 may also converge with classical neurodegenerative pathways, particularly those associated with Alzheimer’s disease (AD). Shared molecular mechanisms include glial reactivity (GFAP, LGALS3), neuroinflammation, BBB disruption, and tau hyperphosphorylation associated with the activation of FK506-binding protein 5 (FKBP5) and glycogen synthase kinase 3 beta (GSK3β) [33,48,51,140,141,154]. Transcriptomic analyses of Long COVID patients have revealed the upregulation of genes involved in proinflammatory and stress-related signaling, such as Kruppel-like factor 4 (KLF4), FKBP5, and LGALS3 [48,51], suggesting a SARS-CoV-2-specific neuroinflammatory signature.

Findings from translational animal models have provided critical insights into the neuropathological consequences of SARS-CoV-2 infection. In murine models, even in the absence of detectable viral RNA in the brain, peripheral SARS-CoV-2 exposure triggers sustained microglial activation, increased expression of proinflammatory cytokines (e.g., IL-6, TNF-α), and impaired hippocampal neurogenesis [56,151,155]. Transcriptomic analyses have revealed the upregulation of neurodegeneration-associated genes, including Ifi204, FKBP5, complement Component 4 (C4), and the C5a receptor (C5aR), which reflect the early molecular patterns observed in Alzheimer’s disease (AD) [56,151,156,157].

Additional studies in K18-hACE2 transgenic mice and hamster models have demonstrated vascular leakage, BBB disruption, astrogliosis, and tau hyperphosphorylation, even in the absence of productive viral replication in the CNS [158,159,160]. Moreover, SARS-CoV-2-infected animals exhibit mitochondrial dysfunction, oxidative stress markers, and alterations in myelin integrity, supporting a functional correlation with cognitive impairment [51,155]. These preclinical findings highlight the capacity of SARS-CoV-2 to induce a sustained neuroinflammatory and neurodegenerative state through immune-mediated and vascular mechanisms. Importantly, such models offer a valuable platform for identifying mechanistic targets beyond classical cytokine pathways, which remain therapeutically limited due to systemic toxicity.

In humans, elevated levels of NFL and GFAP correlate with CNS symptoms, neuroinflammation, and glial activation—hallmarks also observed in early AD [51]. These molecular changes promote a proinflammatory environment that, together with oxidative stress, facilitates tau hyperphosphorylation via activation of GSK3β, ultimately leading to synaptic dysfunction and neuronal loss [51,157].

The upregulation of astrocyte-related factors such as LGALS3 and oxidative stress regulators like KLF4 further supports the existence of a SARS-CoV-2-induced neurodegenerative cascade [48]. Furthermore, vascular pathology constitutes a critical point of convergence between AD and Long COVID. In AD, cerebrovascular dysfunction impairs the clearance of amyloid-β and tau, thereby exacerbating neuronal injury [56,107,108,157]. Similarly, in Long COVID, endothelial damage, cerebral microthrombosis, and disruption of the BBB compromise cerebral perfusion and facilitate the entry of neurotoxic factors into the central nervous system [48,51,73,119]. Neuroimaging findings in both conditions include brain volume loss, white matter abnormalities, and pronounced temporal lobe atrophy [107].

In addition to these structural changes, genetic susceptibility—most notably the apolipoprotein E (APOE) ε4 allele—also modulates the disease trajectory. APOE ε4 carriers, already at elevated risk for AD, have shown increased vulnerability to severe COVID-19 and its neurological sequelae [161,162], likely due to underlying impairments in lipid metabolism, immune regulation, and vascular integrity.

Neuroendocrine dysregulation through HPA axis impairment has been observed in both conditions. Chronic systemic inflammation and psychological stress are associated with hypocortisolism and impaired negative feedback, which perpetuate glial activation and contribute to long-term neuronal injury [142,143,150].

From a therapeutic perspective, this mechanistic overlap highlights opportunities for the development of shared intervention strategies. Natural compounds such as resveratrol, curcumin, and green tea polyphenols have demonstrated antioxidant and mitochondrial-supportive properties through the activation of AMP-activated protein kinase (AMPK), PPARγ, and peroxisome proliferator-activated receptor gamma coactivator 1-alpha (PGC-1α), as well as the nuclear factor erythroid 2-related factor 2 (NRF2) and heme oxygenase-1 (HO-1) signaling pathways [48,51,107,158,163,164]. These neuroprotective mechanisms are often downregulated in both Alzheimer’s disease (AD) and Long COVID [159,164].

In parallel, the proinflammatory transcription factor NF-κB is hyperactivated in SARS-CoV-2 infection, amplifying glial cytokine production. When coupled with impaired NRF2 signaling—normally responsible for controlling oxidative stress and stabilizing inhibitor of NF-κB alpha (IκB-α)—this imbalance drives central nervous system (CNS) homeostasis toward a neurodegenerative state. Dual targeting of NF-κB inhibition and NRF2/PPARγ activation thus emerges as a promising therapeutic approach for both pathologies [159,160,165].

In summary, the overlap between Long COVID and Alzheimer’s disease reflects a complex network of neuroinflammation, vascular dysfunction, mitochondrial impairment, HPA axis dysregulation, and genetic predisposition. Clarifying these shared mechanisms is essential for identifying high-risk individuals and for developing early, targeted neuroprotective strategies to preserve cognitive function following SARS-CoV-2 infection.

## 6. Molecular Markers and Therapeutic Targets

Cognitive impairment in Long COVID appears to result from a multifactorial pathophysiologic process involving persistent neuroinflammation, glial activation, endothelial dysfunction, and compromised neurovascular integrity. Several molecular biomarkers have been associated with these mechanisms. Proinflammatory cytokines such as IL-6 [48,86,88,89,166], IL-1β [48,70,71], and TNF-α [48,83,90] contribute to glial activation, inflammasome signaling, and synaptic loss. Astrocytic and axonal injury is reflected by increased levels of GFAP [33,48,58,74,82] and NFL [33,39,47,48], while FKBP5 and LGALS3 are linked to *tau* phosphorylation and neurotoxicity [48,51].

MicroRNAs, including miR-155, miR-146a, and miR-24, regulate neuroinflammatory cascades, oxidative stress, and blood–brain barrier (BBB) integrity, in part through NF-κB signaling and modulation of NRP1 expression [48,76,77]. Neuroendocrine markers, such as dopamine [136], serotonin [137], and cortisol [142,145], reflect hypothalamic–pituitary–adrenal (HPA) dysfunction and neurotransmitter imbalance. Additional contributions include the stress-related factor KLF4 [49,52] and vascular biomarkers, such as VEGF-A [48,72,74], ICAM-1 [92,104,105,110,111,112,113], VCAM-1 [92,104,105,110,111,112,113], and S100B [92], which are implicated in BBB disruption and microvascular injury. In addition, TGF-β [48,92,106,108] has been linked to both vascular permeability and neurodegenerative processes. These biomarkers, detailed in Table 1, provide insight into disease mechanisms and are promising for diagnosis, patient stratification, and therapeutic targeting.

Persistent systemic inflammation and subclinical myocardial damage may act synergistically to maintain the neurocognitive and multisystem dysfunctions characteristic of Long COVID. A recent study identified elevated levels of IL-6, cardiac troponin, and NT-proBNP, which were significantly associated with an increased risk of mortality and persistent neurological and systemic symptoms [166]. These biomarkers reflect ongoing inflammatory and cardiac stress and may serve as accessible indicators for identifying individuals at risk of long-term cognitive and functional decline. IL-6 plays a central role in neuroimmune interactions and has been associated with microglial activation, BBB disruption, and central nervous system fatigue. Monitoring inflammatory and cardiac biomarkers may therefore be essential for early detection and stratified intervention in patients with neurocognitive manifestations.

Pharmacological approaches currently under investigation target immunomodulation and oxidative stress. IL-6 receptor blockers (e.g., tocilizumab) have demonstrated efficacy in severe COVID-19 and are currently being evaluated for long-term neuroprotection [86,88]. TNF-α inhibitors (e.g., infliximab) reverse microglial synaptic loss in animal models [90], and high TNF-α levels in Long COVID have been correlated with neuropsychiatric symptoms and brain alterations [167]. However, clinical application remains controversial due to concerns about immunosuppression and therapeutic timing [90,91].

Nutraceuticals such as resveratrol, curcumin, and polyphenols may mitigate mitochondrial dysfunction and oxidative stress by activating the AMPK/PPARγ/PGC-1α and NRF2/HO-1 pathways [150]. These compounds exert anti-inflammatory and antioxidant effects, supporting neuronal integrity in both Long COVID and Alzheimer’s disease models [150,152]. Among these, PPARγ and NRF2 are central regulators of the neuroimmune balance. NRF2 improves antioxidant responses, while PPARγ modulates lipid metabolism and inflammation, jointly suppressing NF-κB signaling, which is frequently dysregulated in the brains of patients with Long COVID [165,168].

Targeting this signaling axis may help reduce glial activation, preserve synaptic function, and prevent progressive neurodegeneration. Integrating biomarker data with mechanistic information could guide personalized treatment strategies and mitigate long-term cognitive sequelae in Long COVID [169].

## 7. Conclusions and Perspectives

The cognitive dysfunction observed in Long COVID results from a convergence of pathological mechanisms, including persistent neuroinflammation, endothelial injury, astroglial activation, and neuroendocrine dysregulation. These overlapping processes reflect the early stages of neurodegenerative diseases and may represent an accelerated or amplified vulnerability in predisposed individuals. Circulating and CNS-derived biomarkers, such as IL-6, TNF-α, GFAP, NFL, FKBP5, and LGALS3, reflect neuronal injury, astroglial stress, and systemic immune activation and are emerging as valuable tools for diagnosis, prognosis, and stratification.

Therapeutic approaches under investigation include anti-cytokine agents and molecular modulators of the AMPK/PPARγ and NRF2 pathways, which target key mechanisms of inflammation, oxidative stress, and cellular energy imbalance. These strategies show promise for restoring neuronal resilience and limiting long-term cognitive deficits.

Future research should focus on integrating longitudinal biomarker profiling, high-resolution imaging, and single-cell omics to capture dynamic changes in neuroimmune signaling and vascular integrity. Such multi-layered data may enable early identification of at-risk subgroups and inform precision medicine approaches for cognitive preservation in post-COVID-19 syndromes.

## Figures and Tables

**Table 1:** Molecular biomarkers associated with cognitive dysfunction in Long COVID.

Category	Biomarker	Pathophysiological Role	References
**Fibrotic/** **inflammatory**	TGF-β	Linked to BBB disruption and neurodegeneration	[48,92,106,108]
**Inflammatory**	IL-6	Proinflammatory cytokine; contributes to glial activation and cognitive symptoms	[48,86,88,89,166]
	IL-1β	Mediator of cytokine storm; inflammasome activation	[48,70,71]
	TNF-α	Promotes microglial synaptic loss and neuroinflammation	[37,69,70]
**MicroRNA**	miR-155	Contributes to neuroinflammation through NF-κB signaling	[48,76,77].
	miR-146a	Regulates glial activation and oxidative stress	[48,76,77].
	miR-24	Suppresses NRP1; modulates the BBB and inflammation	[48,76,77].
**Neuroendocrine**	Dopamine	Affects motivation and executive function; deficient in Long COVID	[136]
	Serotonin	Modulates mood, memory, vagal tone; depleted via the kynurenine pathway	[137]
	Cortisol	Reflects HPA axis function; hypocortisolism is linked to fatigue and cognitive impairment	[142,145]
**Neuroinflammatory**	GFAP	Astrocytic injury marker; elevated in both Long COVID and AD	[33,48,58,74,82]
	FKBP5	Associated with tau phosphorylation and neuroinflammation	[48,51].
	LGALS3	Marker of astrocyte-mediated neurotoxicity; implicated in AD and Long COVID	[119]
	NFL	Axonal injury marker; correlates with cognitive symptoms	[33,39,47,48],
**Stress-related**	KLF4	Regulates oxidative stress response and apoptosis	[48,51].
**Vascular**	ICAM-1	Adhesion molecule involved in endothelial activation and leukocyte infiltration	[92,104,105,110,111,112,113]
	VEGF-A	Increases vascular permeability; linked to BBB disruption	[48,72,74]
	S100B	Astrocyte-derived; reflects BBB breakdown and CNS injury	[92]
	VCAM-1	Promotes immune cell adhesion and microvascular injury	[92,104,105,110,111,112,113]

## Data Availability

Not applicable.

## Abbreviations

The following abbreviations are used in this manuscript.

ACE2	Angiotensin-converting enzyme 2
ADEVs	Astrocyte-derived extracellular vesicles
ANS	Autonomic nervous system
APOE-ε4	Apolipoprotein E epsilon 4
ATP	Adenosine triphosphate
BBB	Blood–brain barrier
B-CSF	Blood–cerebrospinal fluid barrier
CAP	Cholinergic anti-inflammatory pathway
CD	Cluster of differentiation
CDC	Centers for Disease Control and Prevention
CNS	Central nervous system
CSF	Cerebrospinal fluid
CXCL	C-X-C motif chemokine ligand
ECs	Endothelial cells
EV	Extracellular vehicles
GFAP	Glial fibrillary acidic protein
GM-CSF	Granulocyte–macrophage colony-stimulating factor
HIF-1α	Hypoxia-inducible factor 1-alpha
HRV	Heart rate variability
ICAM-1	Intercellular adhesion molecule 1
IFN	Interferon
IL	Interleukin
KKS	Kallikrein–kinin system
LGALS3	Galectin-3
miRNAs	MicroRNA
ME/CFS	Myalgic encephalomyelitis/chronic fatigue syndrome
MMSE	Mini-mental state examination
MoCA	Montreal cognitive assessment
MMP-9	Matrix metalloproteinase 9
NFL	Neurofilament light chain
NF-κB	Nuclear factor kappa-light-chain-enhancer of activated B cells
NLRP3	NOD-, LRR-, and pyrin domain-containing protein 3
NRP1	Neuropilin-1
NDEVs	Neuron-derived extracellular vesicles
NRF2	Nuclear factor erythroid 2-related factor 2
PASC	Post-acute sequelae of SARS-CoV-2 infection
PBMCs	Peripheral blood mononuclear cells
POTS	Postural orthostatic tachycardia syndrome
PRRs	Pattern recognition receptors
PPARγ	Peroxisome proliferator-activated receptor gamma
ROS	Reactive oxygen species
SARS-CoV-2	Severe acute respiratory syndrome coronavirus 2
SDMT Symbol	Digit modalities test
S1/S2	Subunit 1/Subunit 2 (spike protein cleavage sites)
S100B	S100 calcium-binding protein β
TGF-β	Transforming growth factor beta
TIAs	Transient ischemic attacks
TMPRSS2	Transmembrane protease serine 2
TNF-α	Tumor necrosis factor alpha
VCAM-1	Vascular cell adhesion molecule 1
VEGF-A	Vascular endothelial growth factor A
VEGFR2	Vascular endothelial growth factor receptor 2
WHO	World Health Organization
